# Mid-Upper Arm Circumference Assessment and Comparison With Weight for Length Z-Score in Infants ≤6 Months as an Indicator of Severe Acute Malnutrition

**DOI:** 10.7759/cureus.18167

**Published:** 2021-09-21

**Authors:** Mahjabeen Zehra, Ali Saleem, Zaubina Kazi, Sadia Parkar

**Affiliations:** 1 Paediatrics and Child Health, Aga Khan University Hospital, Karachi, PAK; 2 Infectious Diseases, Aga Khan University Hospital, Karachi, PAK

**Keywords:** infant, anthropometry, severe acute malnutrition, mid upper arm circumference, whz

## Abstract

Objective

To assess the frequency of severe acute malnutrition (SAM) and to determine the validity of mid-upper arm circumference (MUAC) as compared to weight for length z-score (WLZ-score) as an indicator of the nutritional status in this age group.

Methods

A cross-sectional study, with a purposive sampling was conducted from March 2018 to November 2018 to enroll 540 infants ≤6 months of age from three different sites in Karachi, Pakistan. The anthropometric measurements (MUAC, length and weight) were taken by experienced community health workers. The data were analyzed using SPSS. MUAC was compared with WLZ-score for sensitivity and specificity to observe the concordant among the two diagnostic measures. The Youden Index was used to determine the ideal cut-off for infants less than 6 months of age in this population and the Kappa coefficient was also calculated to assess the agreement between MUAC and WLZ-score.

Results

The study findings revealed that SAM was found in 13.6% (n=74) of the children. MUAC cut-off ≤11.5 cm yielded the Youden Index of 0.31 with 59.5% sensitivity and 71.4% specificity. The total area under receiver operating characteristic curve was 0.70 (95% CI: 0.63, 0.77; P < 0.001). The degree of agreement between mid-upper arm circumference and weight for length z-score to diagnose SAM ranged from 0.2 to 0.3.

Conclusion

The Youden index implied that a MUAC cut-off of ≤11.5 cm can be used as an indicator with acceptable validity for diagnosing SAM in children ≤6 months of age in a low middle income developing country like Pakistan.

## Introduction

There are approximately 3.8 million severely and moderately wasted children under the age of 6 months, mainly in low- and middle-income countries [1]. Moreover, 5.4 million children <5 years die globally each year, with 45% of them directly related to nutrition-related mortality [2]. Current child growth standards use weight for height z scores (WHZ-score) and mid-upper arm circumference (MUAC) measurements as independent measures to identify malnutrition in children between 6 and 60 months of age [3]. It has been argued that these two criteria should be used as complementary measures to independently guide admission and management of malnutrition in this age group [4]. A two-step model has also been put forward suggesting MUAC as a screening tool at the community level, followed by both MUAC and WHZ measurements at the primary health care unit, with both indicators being used independently to diagnose severe acute malnutrition [5].

Despite this suggestion, the conclusions drawn from relevant surveys are not all in agreement. A survey from neighboring India showed that MUAC had low sensitivity (17.5%) and positive predictive value (30.4%) at the recommended cut-off of 11.5 cm for identifying severe acute malnutrition (SAM) in children aged 6-36 months old, arising question on its validity as a screening tool for identifying SAM at the community level [6]. Unlike these results though, another study from India found MUAC cut off of <11.5 cm to be appropriate for identifying children with severe acute malnutrition [7]. A study from Bangladesh also found it acceptable to rely on MUAC alone as a single assessment tool for case finding of SAM in children <5 years of age [8].

On the other hand, MUAC has not been considered a reliable measure for diagnosing severe acute malnutrition in infants ≤6 months old because of a lack of data about its inter-observer reliability [9]. MUAC has been reported though to be a highly efficient and field-friendly tool in identifying SAM children in field surveys as compared to using weight measurements that require standard weight and height/length scale/s, and has been praised for its predictive value for death [10].

In infants under 6 months, the World Health Organization defines SAM as those having a WLZ-score of <-3 standard deviations or the presence of bipedal edema [11]. It has been observed that predictive value of death is better for MUAC measurements than WHZ-score classifications in hospitalized children between 6 months and 5 years of age [9]. MUAC measurements are also less affected by hydration status or edema and are therefore a better instrument of defining acute malnutrition in young infants. MUAC has also been shown to have better reliability than WHZ, as the latter is sensitive to the errors in absolute length and weight measurements.

Availability of adequate data on measurement can make MUAC a simple, affordable and reliable screening tool for severe acute malnutrition in infants up to 6 months of age. This study therefore was conducted with the objective of assessing the frequency of severe acute malnutrition and to determine the validity of mid-upper arm circumference as compared to weight for length z-score as an indicator of the nutritional status in this age group. This study was rationalized to develop MUAC as a standard tool in diagnosing SAM in infants up to 6-month-old by comparing it with the current gold standard WLZ score.

## Materials and methods

This was a cross-sectional observational study conducted in Karachi from March 2018 to November 2018 based on secondary data analysis of an earlier study titled “Building the evidence base for care of acutely ill, undernourished children in limited-resource setting-cohort study” with ethical approval number 4317-Ped-ERC-16. Karachi is located on the southern coastline of Pakistan and is the commercial hub of Pakistan. It is the most populous ethnically diverse city in the country representing communities from all over Pakistan. The study participants in the parent study were enrolled using purposive sampling because of age restriction enrolment in the study, from three sites in Karachi. The sites included, (i) Cattle Colony (Bhains colony), a peri-urban site catering to lower-income families; (ii) Pediatric ward, Civil Hospital Karachi, one of the main public sector hospitals serving a large population pool of lower-income families; (iii) the vaccination center at Aga Khan University, a private tertiary hospital largely population pool of middle to higher-income families. All infants who were under-6 months of age and visiting any of the three study sites for medical checkup with informed consent were included. Pre-term infants and those with severe congenital abnormalities, such as heart defects, neural tube defects and Down’s syndrome, that are associated with growth defaulting were excluded from the study. The rationale of having a hospital, a community and a vaccination center is obtaining the community representative sample.

Under the supervision of a research assistant, all measurements were taken by experienced field workers who had been trained by the principal investigator in anthropometric assessment of weight, length and MUAC. Senior research staff monitored the whole data collection process to protect the integrity of the data collected.

Weight was measured using a digital basin measuring scale (SECA 376). The length was measured by placing the child horizontal on a Shorr board and the MUAC was measured using standard UNICEF MUAC tape in the left arm at a flexed position. Weight was taken once with clothes removed, with two observers. Weight, length and MUAC were each taken twice by independent observers and an average of each value is taken as final measurement. If the inter-observer difference was more than 0.5 cm, measurements were repeated. These measurements were then used to calculate weight for length (WLZ) score, weight for age (WAZ) score and length for age (LAZ) scores using the WHO growth charts and the WHO anthropometry calculator [12].

Sample size was calculated using 33% as the percentage frequency of severe acute malnutrition in Pakistan, with a confidence level of 95% and precision of 4%, to be 531 [13]. Against this sample size, data of total 540 infants were included in the study (180 infants from each site, with 30 infants in each category of 1-month intervals from 1 to 6 months).

Statistical Package for Social Sciences (SPSS) was used for data analysis.MUAC was compared with WHZ for sensitivity and specificity to diagnose severe acute malnutrition. The Youden Index was calculated to determine the ideal cut-off for infants less than 6 months of age. The Kappa coefficient was also calculated to calculate the agreement between MUAC and WLZ for diagnosing severe acute malnutrition.

## Results

The study results showed that out of total of 540 children, aged 1-6 months, 57.2% (n=309) were male and 50.9% (n=275)were on exclusive breastfeeding (n=276). Severe acute malnutrition (WLZ Score <3 S.D.) were found in n=74 (13.7%) of the study population out of which n=52 (70.3%) were from a government hospital, n=12 (16.2%) were from tertiary care hospital while n=10 (13.5%) were from the peri-urban site. The mean weight, length and MUAC at 6 months were 5.35±1.49 grams; 59.51±5.76 cm; and 12.10±1.71 cm respectively (Table 1).

**Table 1:** Demographic characteristics of the study population (n=540).

Site	Count (%)	
Govt. hospital	182 (33.7)	
Peri-urban site	180 (33.3)	
Tertiary care hospital	178 (33.0)	
Age in months	Count (%)	
1	88 (16.3)	
2	91 (16.9)	
3	90 (16.7)	
4	91 (16.9)	
5	90 (16.7)	
6	90 (16.7)	
Gender	Count (%)	
Male	309 (57.2)	
Female	231(42.8)	
Breastfeeding	Count (%)	
Exclusively breastfeeding	275 (50.9)	
Mixed feeding	180 (33.3)	
No	85 (15.7)	
Severe acute malnutrition^1^	74 (14)	
Anthropometry	Mean± SD	Median (IQR)
Weight in kg	5.35 ± 1.49	5.38 (2.08)
Length	59.51 ± 5.76	59.4 (7.9)
Mid-upper arm circumference	12.10 ± 1.71	12.3 (1.8)
Occipitofrontal circumference	38.97 ± 3.00	39.0 (3.6)

**Figure 1:**
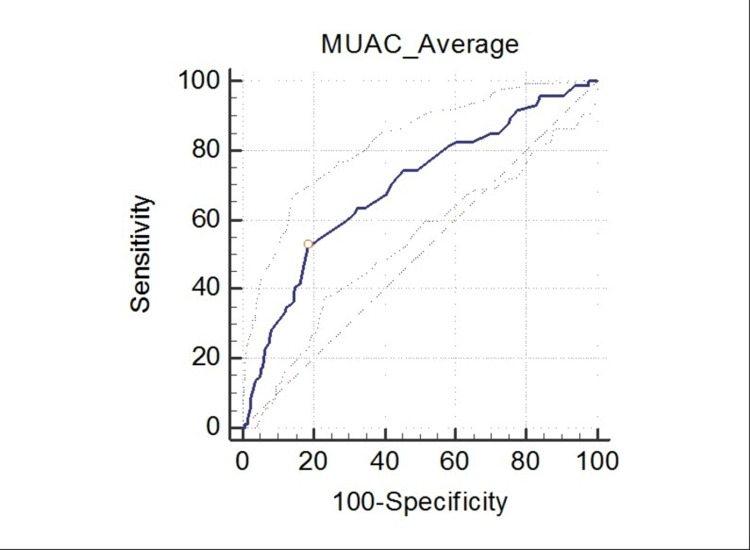
Receiver operating characteristics curve depicting the sensitivity and specificity of MUAC cut off of ≤11.5 cm. MUAC: mid-upper arm circumference.

The study findings further revealed that overall the MUAC cut-off ≤11.5 cm yielded the Youden index of 0.31 with sensitivity and specificity of 59.5% and 71.4% respectively. Moreover, the total area under ROC curve was 0.70 (95% CI: 0.63, 0.77; P < 0.000) (Figure 1).

Moreover, the prevalence of under nutrition as measured by WLZ was found to be highest at the age of 3 months (31.1%) (data not shown).

Among study participants with severe acute malnutrition, the mean MUAC was found to be lowest at the age of 2 months (9.60±1.24 cm) and highest at the age of 5 months (11.76±2.19 cm) (Table 2).

**Table 2:** Nutritional spectrum of study population. MUAC: mid-upper arm circumference; SAM: severe acute malnutrition; WLZ: weight for length score; WAZ: weight for age score; LAZ: length for age score.

	All study population			Malnourished infants as per WHO child growth standards			
	Weight in kg, mean±SD	Length in cm, mean±SD	MUAC in cm, mean±SD	Severe Stunting (LAZ < -3SD) (%, n)	Severe Wasting (WLZ < -3SD) (%, n)	Severe Underweight (WAZ < -3SD) (%, n)	MUAC among SAM infants
1 month	4.05±0.87	53.69±3.23	11.17±1.34	5.7 (5)	14.8 (13)	11.4 (10)	10.50±1.60
2 months	4.59±1.05	56.27± 3.72	11.47±1.57	11 (10)	6.6 (6)	16.5 (15)	9.60±1.24
3 months	5.02±1.12	58.12±4.32	11.95±1.65	16.7 (16)	8.9 (8)	24.4 (22)	10.8±1.83
4 months	5.73±1.27	60.83± 4.23	12.50±1.55	16.5 (15)	16.5 (15)	22.0 (22)	11.18±1.62
5 months	6.09±1.31	63.19± 4.26	12.77±1.82	11.1 (10)	21.1 (19)	21.1 (19)	11.76±2.19
6 months	6.67±1.37	65.31± 4.20	12.88±1.48	10 (9)	14.4 (13)	17.8 (16)	11.34±1.39

The cross-tabulation between WLZ and MUAC for diagnosing severe acute malnutrition showed the sensitivity of MUAC cut-off of ≤11.5 cm to be 59.5% whereas its specificity was found to be 71.4% (Table 3). 

**Table 3:** Cross-tabulation between WHZ and MUAC to diagnose severe acute malnutrition in infants ≤ 6 months old. WHZ: weight for height z scores; MUAC: mid-upper arm circumference; SAM: severe acute malnutrition.

		WHZ		Total
		SAM, n (%)	No SAM, n (%)	
MUAC	SAM	44 (59.5)	132 (28.5)	176
	No SAM	30 (40.5)	331 (71.5)	361
Total		74	463	537

Moreover, the degree of agreement between MUAC and WLZ as shown by Kappa coefficient to diagnose severe acute malnutrition ranged from 0.2 to 0.3 and was highest for 5 months old children (0.37) though still not good or excellent (Table 4). However, it should be recognized that as for other age groups, a proportion of malnourished children will be defined by WLZ or MUAC only.

**Table 4:** Age-based Kappa values showing agreement between MUAC and WHZ to diagnose severe acute malnutrition in infants ≤ 6 months old. WHZ: weight for height z scores; MUAC: mid-upper arm circumference; SAM: severe acute malnutrition.

Age in months	K
3 months	0.220
4 months	0.339
5 months	0.375
6 months	0.232

## Discussion

The study findings revealed that severe acute malnutrition (WHZ score <3 S.D.) was found in 13.6% of the study population. Moreover, the MUAC cut-off ≤11.5 cm yielded the Youden index of 0.31 with sensitivity and specificity of 59.5% and 71.4% respectively to diagnose severe acute malnutrition in infants up to 6 months of age. Furthermore, the degree of agreement between MUAC and WHZ as shown by kappa coefficient to diagnose severe acute malnutrition was slight and ranged from 0.2 to 0.3 in the same age group.

The WHO defines severe acute malnutrition in infants who are 0-5 months of age as either weight-for-length less than -3 Z-score, or the presence of bilateral pitting edema [14]. The use of MUAC as a screening measure for assessing undernutrition has the following advantages: makes use of simple equipment, is easy to carry to field sites, and requires minimal training [15]. However, MUAC is not currently used as a screening tool in children less than 6 months of age. It has been documented that MUAC is equally effective, if not better, as a diagnostic tool in identifying children with a high risk of death as it was found to have the highest ROC curve in comparison to other nutritional indices [16]. WHO, as per the integrated community case management guidelines, recommends the use of MUAC for assessing and treating sick children in the community [17].

The study results showed that MUAC cut off of ≤11.5 cm can be used with reasonable sensitivity and good specificity to diagnose severe acute malnutrition in infants up to 6 months of age. A study from South Africa in 2015 reported that weight for height measurements identified 7.7% of children as malnourished as compared to 6.6% by MUAC in 0 to 59 month age group, concluding weight for height measurements as more sensitive than MUAC for diagnosing acute malnutrition, but at the same time recommending further studies using both indicators at the community level for diagnosing moderate and severe malnutrition in infants aged 0 to 6 months [18].

The study results further revealed a slight degree of agreement between MUAC and WHZ to diagnose severe acute malnutrition in infants up to 6 months of age as shown by a kappa coefficient that ranged from 0.2 to 0.3. The African study cited above also reported a kappa statistic value of 0.27 constituting a slight agreement between WHZ and MUAC in children aged 0 to 59 months [18]. This similarity indicates that an agreement does exist between WHZ and MUAC for diagnosing severe acute malnutrition in this age group and therefore lends a degree of credibility to our study results.

Unfortunately in our local and regional context, to the best of our knowledge, any relevant published data for a meaningful comparison with our study findings is almost non-existent. Unless the evidence base is broadened and MUAC as a screening and diagnosing tool for severe acute malnutrition in young infants is rigorously compared with current standards and its pros and cons are weighed carefully, questions on its potential usage as an indicator of severe acute malnutrition in young infants will remain unanswered.

Developing a cut-off for MUAC for diagnosing SAM in infants 0-6 months may be beneficial as MUAC cut-offs of ≤11.5 cm and ≤11.0 cm have earlier been shown to effectively identify infants between the ages of 6-14 weeks with increased risk of death (HRs 4.5, 95% CI: 1.4-15 and 9.5, 95% CI: 2.6-35 respectively) [9]. Literature also suggests that MUAC can be more reliably measured than weight for length as a diagnostic measure for severe acute malnutrition by community health workers (Intra-class correlation 0.96 vs. 0.71 respectively) [19].

It is acknowledged that this study has certain limitations. The prime limitations of the study were non-exclusion of small for gestational age infants from the study along with the use of purposive sampling method. Moreover, the applicability of MUAC as an indicator of childhood malnutrition is limited as children grow rapidly in the first 6 months of life and more specific cut-offs may be needed for children under 3 months of age. Furthermore, evidence does not suggest occurrence of wasting below two weeks of age so the results cannot be generalized to that population as well.

## Conclusions

The MUAC cut-off of ≤11.5 cm yielded a Youden Index with reasonable sensitivity and good specificity implying that MUAC can be used as an indicator with acceptable validity for diagnosing childhood malnutrition children up to 6 months of age in low middle income developing country like Pakistan that has a moderate prevalence of stunting in this age group. It is recommended that the usefulness of MUAC as an indicator to predict malnutrition-associated outcomes other than SAM such as the risk of infections, morbidity and mortality in infants ˂ 6 months of age should be assessed in future studies.
